# Supplementary material for: Modeling glioblastoma heterogeneity as a dynamic network of cell states
Source: Mol Syst Biol. 2021 Sep 16;17(9):e10105. doi: 10.15252/msb.202010105 (PMC8444284; doi:10.15252/msb.202010105)
Supplement: Supplementary file 6 — Source Data for Figure 5 [file MSB-17-e10105-s004.zip › Figure5A_sourcedata/GSEA_3071/hallmarks_stateII.GseaPreranked.1623742536692/HALLMARK_TGF_BETA_SIGNALING.html]

Details for gene set HALLMARK\_TGF\_BETA\_SIGNALING[GSEA]

|  || Dataset | state23071 |
| Phenotype | NoPhenotypeAvailable |
| Upregulated in class | na\_neg |
| GeneSet | HALLMARK\_TGF\_BETA\_SIGNALING |
| Enrichment Score (ES) | -0.3750123 |
| Normalized Enrichment Score (NES) | -1.5578834 |
| Nominal p-value | 0.05027933 |
| FDR q-value | 0.15150242 |
| FWER p-Value | 0.623 |
Table: GSEA Results Summary

  

Fig 1: Enrichment plot: HALLMARK\_TGF\_BETA\_SIGNALING      
 Profile of the Running ES Score & Positions of GeneSet Members on the Rank Ordered List

  

| PROBE | GENE SYMBOL | GENE\_TITLE | RANK IN GENE LIST | RANK METRIC SCORE | RUNNING ES | CORE ENRICHMENT || 1 | SMURF2 |  |  | 224 | 0.851 | -0.0002 | No |
| 2 | SERPINE1 |  |  | 299 | 0.756 | 0.0303 | No |
| 3 | THBS1 |  |  | 624 | 0.568 | -0.0126 | No |
| 4 | ID1 |  |  | 769 | 0.509 | -0.0152 | No |
| 5 | NOG |  |  | 1031 | 0.433 | -0.0515 | No |
| 6 | PPP1R15A |  |  | 1284 | 0.381 | -0.0888 | No |
| 7 | SLC20A1 |  |  | 1400 | 0.357 | -0.0940 | No |
| 8 | BCAR3 |  |  | 1454 | 0.347 | -0.0847 | No |
| 9 | KLF10 |  |  | 1662 | 0.315 | -0.1153 | No |
| 10 | HDAC1 |  |  | 1915 | 0.283 | -0.1589 | No |
| 11 | PPP1CA |  |  | 1954 | 0.279 | -0.1503 | No |
| 12 | XIAP |  |  | 2355 | -0.265 | -0.2315 | No |
| 13 | BMPR1A |  |  | 2800 | -0.335 | -0.3190 | No |
| 14 | HIPK2 |  |  | 3029 | -0.379 | -0.3506 | Yes |
| 15 | ID3 |  |  | 3112 | -0.402 | -0.3448 | Yes |
| 16 | TGFBR1 |  |  | 3139 | -0.411 | -0.3247 | Yes |
| 17 | ARID4B |  |  | 3330 | -0.467 | -0.3413 | Yes |
| 18 | PMEPA1 |  |  | 3390 | -0.494 | -0.3239 | Yes |
| 19 | APC |  |  | 3422 | -0.510 | -0.2987 | Yes |
| 20 | SPTBN1 |  |  | 3515 | -0.551 | -0.2858 | Yes |
| 21 | SKIL |  |  | 3531 | -0.560 | -0.2534 | Yes |
| 22 | ID2 |  |  | 3664 | -0.654 | -0.2437 | Yes |
| 23 | BMPR2 |  |  | 3715 | -0.698 | -0.2110 | Yes |
| 24 | SMAD1 |  |  | 3719 | -0.699 | -0.1667 | Yes |
| 25 | TGIF1 |  |  | 3728 | -0.707 | -0.1230 | Yes |
| 26 | CTNNB1 |  |  | 3753 | -0.731 | -0.0818 | Yes |
| 27 | RAB31 |  |  | 3757 | -0.737 | -0.0351 | Yes |
| 28 | CDKN1C |  |  | 3821 | -0.809 | 0.0016 | Yes |
| 29 | WWTR1 |  |  | 3936 | -1.027 | 0.0398 | Yes |
Table: GSEA details [plain text format]

  

Fig 2: HALLMARK\_TGF\_BETA\_SIGNALING: Random ES distribution      
 Gene set null distribution of ES for **HALLMARK\_TGF\_BETA\_SIGNALING**
